# Culturing Pancreatic Islets in Microfluidic Flow Enhances Morphology of the Associated Endothelial Cells

**DOI:** 10.1371/journal.pone.0024904

**Published:** 2011-09-22

**Authors:** Krishana S. Sankar, Brenda J. Green, Alana R. Crocker, Jocelyne E. Verity, Svetlana M. Altamentova, Jonathan V. Rocheleau

**Affiliations:** 1 Institute of Biomaterials and Biomedical Engineering, University of Toronto, Toronto, Ontario, Canada; 2 Department of Physiology, University of Toronto, Toronto, Ontario, Canada; 3 Toronto General Research Institute, University Health Network, Toronto, Ontario, Canada; University of Bremen, Germany

## Abstract

Pancreatic islets are heavily vascularized *in vivo* with each insulin secreting beta-cell associated with at least one endothelial cell (EC). This structure is maintained immediately post-isolation; however, in culture the ECs slowly deteriorate, losing density and branched morphology. We postulate that this deterioration occurs in the absence of blood flow due to limited diffusion of media inside the tissue. To improve exchange of media inside the tissue, we created a microfluidic device to culture islets in a range of flow-rates. Culturing the islets from C57BL6 mice in this device with media flowing between 1 and 7 ml/24 hr resulted in twice the EC-density and -connected length compared to classically cultured islets. Media containing fluorescent dextran reached the center of islets in the device in a flow-rate-dependant manner consistent with improved penetration. We also observed deterioration of EC morphology using serum free media that was rescued by addition of bovine serum albumin, a known anti-apoptotic signal with limited diffusion in tissue. We further examined the effect of flow on beta-cells showing dampened glucose-stimulated Ca^2+^-response from cells at the periphery of the islet where fluid shear-stress is greatest. However, we observed normal two-photon NAD(P)H response and insulin secretion from the remainder of the islet. These data reveal the deterioration of islet EC-morphology is in part due to restricted diffusion of serum albumin within the tissue. These data further reveal microfluidic devices as unique platforms to optimize islet culture by introducing intercellular flow to overcome the restricted diffusion of media components.

## Introduction

Pancreatic islets are highly vascularized micro-organs designed to effectively sense glucose and secrete insulin directly into the bloodstream. The characteristically dense vasculature of islets is comprised of endothelial cells (ECs) that are thin and highly fenestrated, and therefore directly connect the insulin secreting beta-cells to the blood [1]. Islets maintain this vasculature once harvested for *ex vivo* studies. However, it is well established that during *ex vivo* culture, the density of intra-islet ECs decreases approximately 50% in the first day and is almost completely gone by day four [2]. This loss of ECs during culture severely limits long-term study of the interaction between beta-cells and ECs in the tissue, and is directly relevant to the treatment of type 1 diabetes due to the critical role of the ECs from donor islets in the revascularization of this tissue during transplantation [2], [3], [4]. The final reconnecting vessels are formed by EC from the donor and recipient, which makes it vital to maintain these cells prior to transplantation [2], [4]. Our goal was therefore to delay the loss of islet-ECs during culture.

Islets isolated from the pancreas are severed from the vasculature and once in culture rely on diffusion for nutrient supply and by-product removal. We postulate that the ECs inside islets in culture die in part due to an absence of blood flow to provide media exchange. It is well established that ECs in culture undergo apoptosis when serum is reduced in the culture medium [5]. However, in isolated islets, serum proteins such as albumin will not diffuse significantly into the tissue due to large hydrodynamic radius and transient binding properties [6]. Alternatively, any nutrients that are consumed (eg. O_2_, glucose) or products that are secreted (eg. insulin, lactate) will form gradients that may adversely affect the ECs. Overall, limited media exchange during islet culture can have detrimental effects on the ECs within the islet. It should also be noted that flow-induced shear can have a positive effect on ECs, acting as a biomechanical stimulus and anti-apoptotic signal [7], [8], [9]. Conversely, flow can negatively affect epithelial cells [10], and may therefore negatively impact glucose-stimulated insulin secretion.

To test the effect of fluid flow on maintenance of islet ECs, we developed a hemodynamic environment using a custom-designed microfluidic device. Our devices place islets in laminar flow, similar to devices previously used to monitor bulk islet O_2_-consumption rate using perfusion column chambers [11], [12], [13]. In contrast to perfusion column designs, microfluidic devices require higher pressure-drops to induce flow [14] and are completely compatible with fluorescence microscopy [14], [15]. We aimed to use these devices combined with fluorescence microscopy to treat islets with media flow and image the effect on ECs and beta-cells.

## Materials and Methods

### Microfluidic Device Fabrication

Devices were fabricated using elastomer polydimethylsiloxane (PDMS) (Dow-Corning) as described previously [16], [17]. The cured mould was irreversibly bonded to a 24×50-mm coverslip (VWR Scientific) by oxygen plasma treatment (Harrick Scientific, Ossining, NY) and Tygon tubing was inserted directly into the cored-out port holes.

### Microfluidic Device Design

The microfluidic device design and process are described in detail in the Supplemental Material (**Text S1 & Fig. S1, S2, and S3**). Briefly, islets were brought into 125 µm tall ×300 µm wide microfluidic channels through inlet port tubes. Islets moved along these channels by media flow until they reached a dam wall. This dam structure was 25 µm tall, 1,800 µm wide, and 0.13 cm long, and allowed solution to flow past but blocked the movement of islets. Microfluidic devices provide only non-turbulent or laminar flow at the flow rates used in these studies [14]. Two main designs were used for these studies: a three-channel microfluidic device and an independent-channel microfluidic device (**Fig. S1**). The three-channel device placed islets in three different flow-rates based on varied channel lengths and by using a single syringe pump. The independent-channel device provided a single flow-rate in parallel channels to facilitate subsequent live cell imaging or effluent collection.

### Ethics Statement

Animal procedures were approved by the Animal Care Committee of the University Health Network, Toronto, Ontario, Canada in accordance with the policies and guidelines of the Canadian Council on Animal Care (Animal Use Protocol #1531).

### Pancreatic Islet Isolation and Treatment

Pancreatic islets were isolated from 8- to 12- week-old C57BL6 male mice by using collagenase digestion (Roche) [18], [19]. Islets were cultured in RPMI medium 1640 containing 11 mM glucose, 10% FBS, 5 U/ml penicillin-streptomycin, and 20 mM HEPES. Control islets were incubated in a non-treated culture dishes in a humidified incubator at 37°C and 5% CO_2_. Device-treated islets were loaded into the microfluidic devices shortly after isolation and incubated in a custom-built desk-top incubator described in detail in the Supplemental Material (**Fig. S2**). Briefly, the microfluidic device was submerged in a stirred water bath at 37°C with flow driven by a syringe pump. The reservoir media was also submerged in a separate water-bath maintained just above 37°C to reduce formation of air bubbles in the microfluidic channel. A cap of mineral oil was placed on top of the media in the reservoir to reduce evaporation and drift in pH.

### Immunofluorescent Detection

Control and device-treated islets were labelled within the microfluidic device. Islets were fixed (2% PFA, PBS, 1 hr, 100 µL/hr), blocked (PBS, 0.1% TritonX-100, 4°C, 10% Normal Goat Serum, 4 hr, 15 µL/hr), incubated with primary antibody (PBS, 0.1% TritonX-100, 4°C, 1% Normal Goat Serum, 1∶100 dilution rat anti-mouse PECAM-1, 3 hr, 3 µL/hr), rinsed (PBS, 0.1% TritonX-100, 4°C, 10% Normal Goat Serum, 1 hr, 15 µL/hr) and incubated with 1∶500 goat anti-rat Alexa 633 (PBS, 0.1% TritonX-100, 4°C, 10% Normal Goat Serum, 3 hr, 15 µL/hr). Islets were imaged using a 20×0.50 NA objective lens in 3 µm steps for 16–20 slices (dependant on the size of the islet) on an Olympus FluoView 300 microscope.

Images were analyzed using ImageJ 1.41o (Wayne Rasband, NIH, USA). EC fractional area was measured by calculating the area of PECAM-1 labelling divided by the total area of the islet (6 slices/islet). Total connected length was determined using Simple Neurite Tracer throughout the entire stack of images. PECAM-1 labelled regions were tracked through the stack and the resulting summation was normalized to the circumference of the islet. The average of individual islets was used for statistical analysis. A two tail, unpaired, two sample student's t-test was used and significance was determined with p<0.05 (*) and p<0.01 (**).

### Islet intracellular calcium

Islets were preincubated with either 4 µM of Fura-2 or Fluo-4 in imaging buffer supplemented with 2 mM glucose for 1–2 hr prior to imaging. Fura-2 images were collected using the 40×1.3 NA oil immersion lens of an Eclipse Ti-S inverted microscope with 340 nm and 380 nm excitation and 510±40 nm emission filter (PTI EasyRatioPro System, USA). Fluo-4 images were collected on an LSM710 Microscope, 20×0.80 NA objective lens, 488-nm laser line, and 493–630 nm emission. The device and its reagents were placed on the microscope stage inside a 37°C stage-top incubator (Okolabs).

### Two-photon NAD(P)H Imaging

NAD(P)H imaging was done as previously described using a 40×1.3 NA oil immersion objective lens and internal detector (385–550 nm) of a LSM710 microscope (Zeiss) [20], [21]. The Ti∶Saph laser was tuned to 705 nm and attenuated to ∼3 mW at the sample (Coherent).

### Insulin Secretion

Islets in a single channel were stimulated with 200 µL/hr flow and 37°C. Islets were subsequently treated for one hour with 2 mM and 11 mM glucose, each providing 200 µL of effluent. The flow rate was increased to 200 µL/min and 1% TritonX-100 introduced to release the total insulin content. The effluents were diluted 50×(2 and 11 mM glucose) and 200×(1% TritonX-100) prior to insulin ELISA detection.

## Results

### A Microfluidic Device to Mimic *in vivo* Hemodynamics

**Figure 1:**
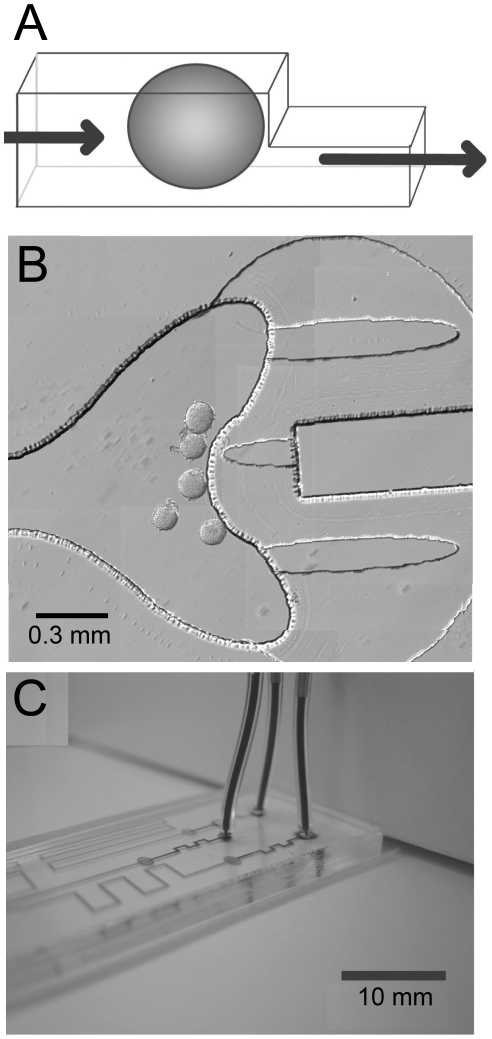
A microfluidic device to treat pancreatic islets with laminar flow. (A) A schematic of a microfluidic channel is shown to demonstrate how pancreatic islets are held in laminar flow. Pancreatic islets (*oval*) are brought into microfluidic channels (*outlined*) and trapped when the channel height drops from 125 to 25 µm. This stops the islet from moving down the channel while allowing fluid to flow past the islet (*arrows*). (B) A representative image of islets held at the ‘pea pod’ shaped dam in a channel. In this experiment, the islets were cultured for 24 hr prior to loading. The islets are sitting in the tall channel (125 µm) against the dam wall to the right. Flow in the device is past the islets from left to right. (C) An image of the three channel microfluidic device used for varying fluid flow-rates past islets. The device shown is filled with bromophenol blue solution to highlight the channels and tubing. The device has three individual inputs (*tubing*) with a common output (not shown). The three microfluidic channels (125 µm) each have a dam for islet retention (*spherical region*, 25 µm) followed by varying lengths of output channels (125 µm). These varied channel lengths allowed media flow to be controlled with one syringe pump, and media flow at three different flow-rates relative to length. Flow rates were ultimately determined by measuring the height of the input reservoirs before and after incubation.

Islets placed in culture no longer experience blood flow. To introduce intracellular flow, we developed a microfluidic device for long-term culture (**Fig. 1** **& S1**). Similar to our previous microfluidic device designs, islets were trapped at a channel dam (**Fig. 1A & B**) [22]. The dam was fashioned by dropping the channel height from 125 to 25 µm, effectively preventing islets from moving further down the channel while still allowing fluid to flow. The device shown had three channels of varying length with individual inputs and one common output (**Fig. 1C**). This design allowed us to culture islets in three different flow-rates using a single syringe pump.

**Figure 2:**
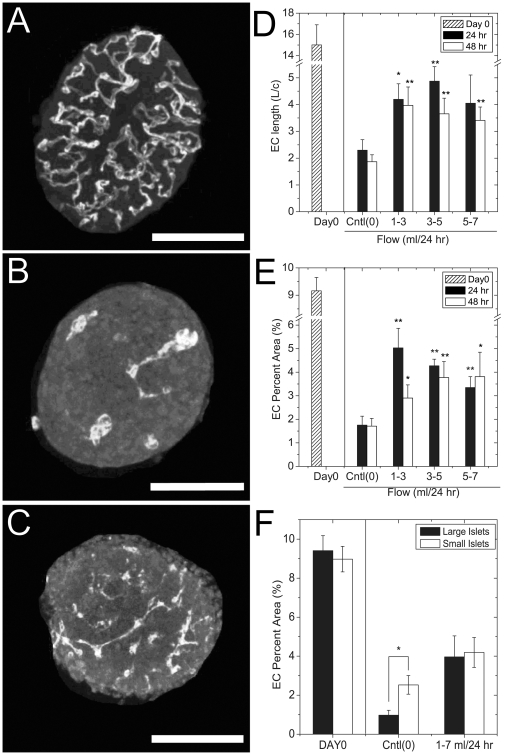
The effect of fluid-flow on EC area and connected length. Pancreatic islets were cultured in either the microfluidic device with flow (device-treated) or in non-flowing media in an incubator (*control*) followed by anti-PECAM-1 immunolabelling. (A) A representative Day 0, freshly isolated islet shows heavy vascularization of the tissue prior to culture. (Scale bar 125 µm) (B) A representative control islet cultured in no-flow for 24 hr displaying characteristic coalescence of EC. (Scale bar 100 µm) (C) A representative islet cultured for 24 hr in a microfluidic device displaying increased EC density and connected length compared to control islets (*Cntl(0)*). (Scale bar 150 µm) (D) The total EC connected length relative to the circumference (*EC Length L/c*) of freshly isolated islets (*hatched bar*), control islets (*Cntl(0)*) at 24 (black bars) and 48 hr (open bars), and device-treated islets exposed to fluid flow (1–3, 3–5, and 5–7 ml/24 hr). (E) The EC area relative to islet area (*EC percent area*) of freshly isolated islets (*hatched bar*), control islets (*Cntl(0)*) at (24 (black bars) and 48 hr (open bars), and device-treated islets exposed to fluid flow (1–3, 3–5, and 5–7 ml/24 hr). The EC length and fractional areas were measured from the islets isolated from at least 3 different mice on independent days. The data shown is the mean ± sem for the following number of pooled islets: 43 (Day0), 14 (Control, 24 hr), 12 (24 hr, 1–3 ml/24 hr), 19 (24 hr, 3–5 ml/24 hr), 10 (24 hr, 5–7 ml/24 hr), 16 (Control, 48 hr), 10 (48 hr, 1–3 ml/24 hr), 7 (48 hr, 3–5 ml/24 hr), and 7 (48 hr, 5–7 ml/24 hr). (F) The EC fractional area for the data shown in Fig. 2E re-grouped into large and small islets based on the median diameter (178 µm). Data shown are the mean ± sem for the following number of pooled islets: 21 (Large Islets, Day0), 22 (Small Islets, Day(0)), 9 (Large Islets, Cntl(0)), 8 (Small Islets, Cntl(0)), 12 (Large Islet, 1–7 ml/24 hr), and 12 (Small Islets, 1–7 ml/24 hr). The * and ** indicate p<0.05 and p<0.01 compared to control (*Cntl(0)*) or where otherwise indicated.

To measure the effect of time (0, 24, and 48 hr) and flow rate (no flow and flow between 1 and 7 ml/24 hr) on EC morphology, islets cultured in dishes (no flow) or in the three channel microfluidic device were assessed by immunofluorescence imaging of PECAM-1 (**Fig. 2**). These flow rates were chosen to approximate a physiologically relevant range of shear stress (1–14 dyn/cm^2^) based on the device geometry [23]. In comparison, individual rat islets *in vivo* receive ∼0.03 ml/24 hr of blood flow [24], [25]. Islets fixed immediately post-isolation were heavily vascularised with EC that branched throughout the islet (**Fig. 2A**). The morphology of the ECs in control dish-cultured islets changed significantly in 24 hr, appearing to coalesce (**Fig. 2B**) [2]. In contrast, the ECs of islets treated in the device with flow had greater density and vascular structure throughout the islet compared to control (**Fig. 2C**). To quantify these changes in morphology, we measured the average relative length and fractional area of the ECs in a number of islets (**Fig. 2D–F**). After 24 and 48 hr of culture, the length and area was significantly decreased in both control and device-treated islets compared to freshly isolated islets (**Fig. 2D & E** **, Day0 vs. Cntl(0)**). However, we observed significantly denser and longer ECs in islets cultured in the device at each time point compared to control islets. We also measured a dramatic decline in EC density at extreme flow rates (0.98±0.08 and 1.16±0.10 fold-EC fractional area at 0.3 and 30 ml/24 hr compared to control, respectively) consistent with an envelope of optimal flow rate. Even though microfluidic devices are inherently permeable to oxygen [26] and we are flowing at rates normally experienced *in vivo* (0.03 ml/24 hr/islet) [24], [25], we may be limiting nutrient supply (glucose, O_2_) at 0.3 ml/24 hr. We may also be inducing shear damage to the tissue at 30 ml/24 hr. To explore the effect of islet size on EC morphology, we re-examined the data shown in Figure 2E based on islet diameter (**Fig. 2F**). Grouping the data from large and small islets (defined as above and below the median islet diameter (178 µm), respectively), we observed no difference in EC fractional area of freshly isolated islets (Day0). After 48 hrs of culture in the absence of flow, large islets had significantly lower EC fractional area than small islets. These data are consistent with a more significant loss of EC morphology in large islets. In contrast, culturing islets in flow (1–7 ml/24 hr) resulted in no difference in EC fractional area between large and small islets, consistent with flow-rate rather than islet size determining EC morphology. Overall, these data show that islets treated to media flow in the device (1–7 ml/24 hr) maintained more than twice their EC density and connected length when compared to islets traditionally cultured in the absence of flow.

### The Mechanism of Enhanced Morphology

**Figure 3:**
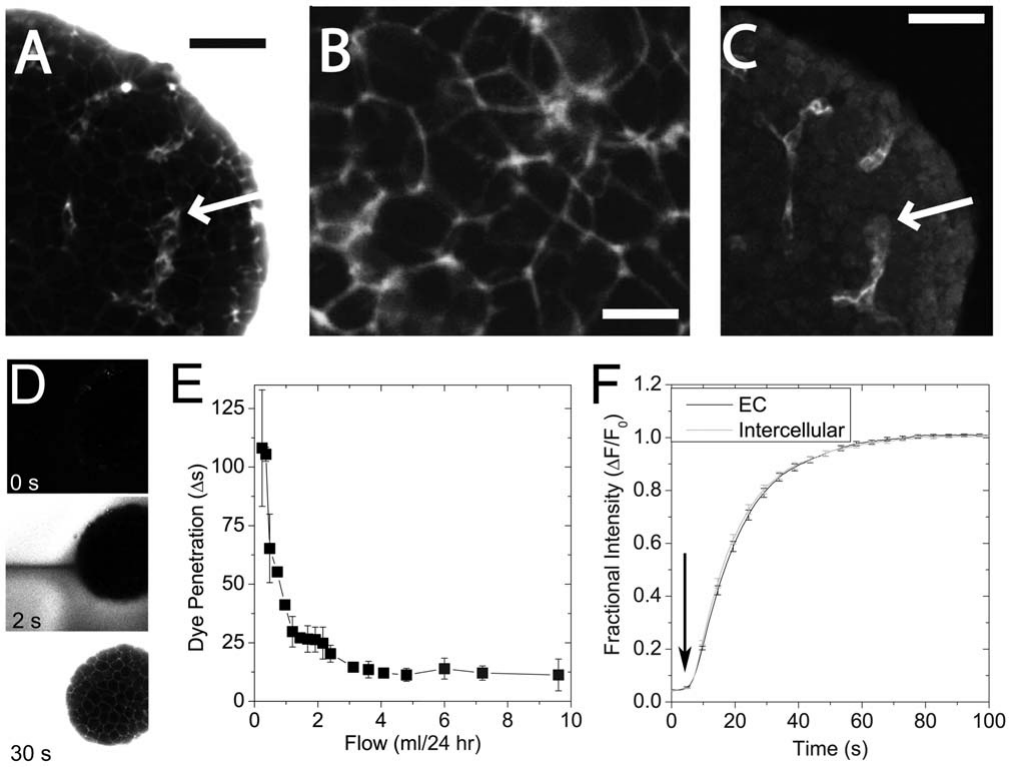
Media access in a microfluidic device measured using fluorescent dextran. To explore the nature of the fluid flow inside treated islets, real-time fluorescent imaging was applied to 24 hr device-treated islets (3 ml/24 hr). (A) We flowed fluorescent dextran (3,000 MW) into the microfluidic device and imaged penetration of this dye into the tissue. The dye fills the channel and intercellular spaces of the islet. Connected (path-like) regions that resemble islet-EC morphology (*arrow*) were immediately evident (black scale bar = 40 µm). (B) A magnified image shows that fluorescent dextran surrounds each cell in the islet (Scale bar = 15 µm). (C) These same islets were immunofluorescently labelled for ECs using PECAM-1 immunofluorescence. The connected regions identified by fluorescent dextrans co-labelled as ECs (compare arrows in A and C) (Scale bar = 40 µm). (D) To measure the kinetics of fluorescent dextran exchange with the intercellular space, we examined the arrival of the dye as it was introduced into the channel. A typical time series is shown with media flowing 3 ml/24 hr from the right to left. The images shown were acquired at 0, 12, and 101 s after the arrival of fluorescent dextran to the islet until it eventually fills both the channel and intercellular space. In the absence of dye, the channel and islet are dark (0 s). At 12 s after the dye arrival, the channel had filled with dye except for a tail of non-labelled solution pushed from the unlabelled islet. At 101 s after the dye arrival, the intercellular spaces were clearly visible and the dye completely filled the channel. (E) To determine the dependence of media exchange on flow rate, we measured the time required to penetrate the islet at various flow rates. These data were collected from the islets of 4 separate mice on different experimental days. The data is plotted as mean clearance time ± sem for the pooled islet data. (F) Islets were cultured in non-flowing media for less than 24 hr prior to imaging. Fluorescent dextran images were collected in a sequence similar to (D), but with spatial resolution similar to (A). This imaging sequence allowed us to measure the intensity of fluorescent dextran at EC structures (EC, black line) or neighbouring intercellular spaces (Intercellular, grey line). The arrow indicates the arrival of fluorescent dextran at the islets. The fractional intensity was determined from the intensity at time zero and the maximal intensity reached at each region at >100 s. Data are shown from the average ± sem for 90 neighbouring pairs of regions from 7 different islets. No difference in arrival rate was measured between EC and intercellular spaces indicating no preference for flow through the lumen of the ECs.

To determine where media gained access in the tissue, we cultured islets in flow (3 ml/24 hr) for 24 hr and subsequently imaged media supplemented with fluorescent dextran (**Fig. 3A & B**). These and subsequent studies were done using a single channel microfluidic device with on-chip reservoir, which allowed us to quickly exchange the media (**Fig. S3**). Fluorescent dextran does not enter living cells, and therefore reports on the volume of media in the intercellular space. We observed media surrounding each individual cell as well as filling connected pathways that had similar shape to ECs (**Fig. 3A & B**). By fixing and staining these same islets for PECAM-1, we further confirmed that the connected pathways corresponded to the ECs of the islet (**Fig. 3A & C** **,** ***arrows***). These data show that media can access the cells in the center of the islet; however, our PECAM-1 data (**Fig. 2**) suggested that this access is limited at slower flow rates (0.3 ml/24 hr). To examine the flow-rate dependence of media penetration, we measured the time to fill the intercellular space at different flow rates (**Fig. 3D & E**). A representative image series is shown of a single islet as fluorescent dextran is introduced at a flow rate of 3 ml/24 hr from right to left. In this setup, we measured the time between dye front arrival (as dye surrounds the islet) and dye filling the intercellular space. The top panel of Fig. 3D is the image frame immediately prior to the arrival of dye at the islet (0 s). At this time the media and islet are both dark reflecting the absence of dye. At 2 s the dye completely filled the channel, but the islet remained dark indicating the dye had not significantly penetrated the tissue. The visibly dark flow tail streaming behind the islet (at left) occurs due to non-fluorescent media being pushed from the intercellular space. By 30 s, the dye completely filled the intercellular space of the islet and the clear flow stream disappeared. Data collected from multiple islets at multiple flow rates shows that the time for dye to penetrate the islet is exponentially dependent on flow rate (**Fig. 3E**). These data suggest that at the flow rates previously shown to enhance EC morphology (3–6 ml/24 hr), media exchange occurs in less than 30 s. In contrast, at flow rates that did not enhance EC morphology (0.3 ml/24 hr) media exchange was a least 5× slower (>150 s). Importantly, this graph is asymptotic as it approaches 0 ml/24 hr, indicating that in normal static culture the exchange rate due to diffusion is likely much slower (min - hrs). We also re-grouped the data based on islet size, but observed no differences in dye penetration at any of the flow rates between large and small islets. This result was consistent with our previous data showing a similar effect of flow on the morphology of ECs. These data are also consistent with dye penetration being more a function of flow rates in the microfluidic device than of islet size. To determine the route of flow, we also measured fluorescent dextran arrival in neighbouring intercellular and EC-regions spaces in a number of islets (**Fig. 3F**). These data show identical arrival times indicating flow in the device is not preferentially driven through the EC lumen, but rather simply through intercellular spaces.

**Figure 4:**
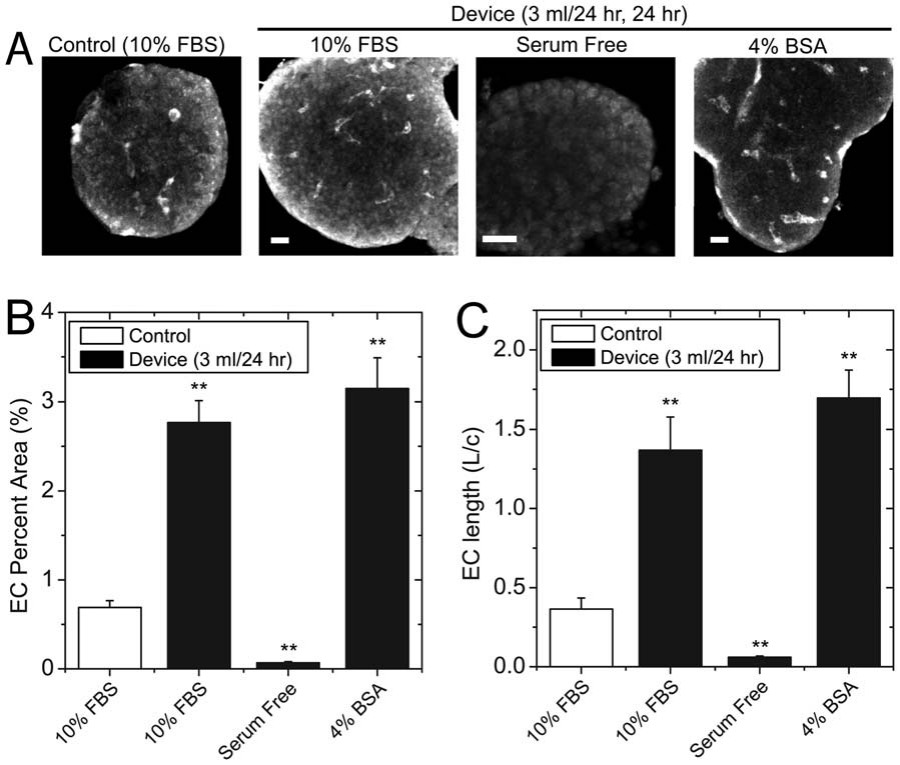
Effect of serum albumin on EC morphology. Islets were cultured for 24 hr in normal culture (Control, 10% FBS) and the microfluidic device (3 ml/24 hr flow rate). The media used in the device was either normal media (10% FBS), serum free media (Serum Free) or serum free media containing 4% bovine serum albumin (4% BSA). The islets were then fixed and immunofluorescently labelled for PECAM-1. (A) Representative maximum projection image of PECAM-1 immunofluorescence for each culture. (B) EC Percent Area of control islets (open bar) and device treated islets (black bars) cultured as indicated. (C) The relative EC length of control islets (open bar) and device treated islets (black bars) cultured as indicated. Each bar consists of the data collected from 10 to 17 islets collected from 3 mice, with the islets from each mouse isolated on independent days. The * and ** indicate p<0.05 and p<0.01 compared to control (*open bar, 10% FBS*), respectively by two-tailed student's t-test.

Serum albumin is a potent anti-apoptotic signal to endothelial cells [5] that due to large hydrodynamic radius has restricted diffusion into tissues [6]. To determine whether enhanced EC morphology in microfluidic flow is due to improved access of serum albumin, we compared islets cultured for 24 hr in a dish (control, 10% FBS) to islets a microfluidic device (3 ml/24 hr). The device treated islets were maintained in media with normal serum (10% FBS), no serum (Serum Free), or bovine serum albumin (4% BSA) (**Fig. 4**). Representative images (**Fig. 4A**) and the quantified morphology (**Fig. 4B & C**) again show increased EC fractional area and length in device treated islets compared to control when cultured in normal serum (10% FBS). In contrast, EC fractional area and length were significantly decreased compared to control in the absence of serum (Serum Free). Addition of bovine serum albumin (4% BSA) resulted in a significant increase in EC fractional area and length compared to control islets and similar responses compared to device-treated islets with normal serum. These data show that the improvement in islet-EC morphology normally observed with in microfluidic flow is due in part to enhanced access of serum albumin to the center of the tissue.

### Beta-cell Function

We measured beta-cell function in multiple ways to determine the effect of flow. Large islets in culture often develop a necrotic core visible under the white light of a microscope. We therefore counted the number of necrotic centers observed in all of our experiments after 48 hrs of culture. Of the 114 islets cultured in a dish, 10 (or 8.8%) developed a necrotic core. In contrast, we did not see a single necrotic core in 50 islets cultured at 3 ml/24 hr for 48 hr. These data are consistent with improved penetration of media to the center of islets when treated with flow in the device.

**Figure 5:**
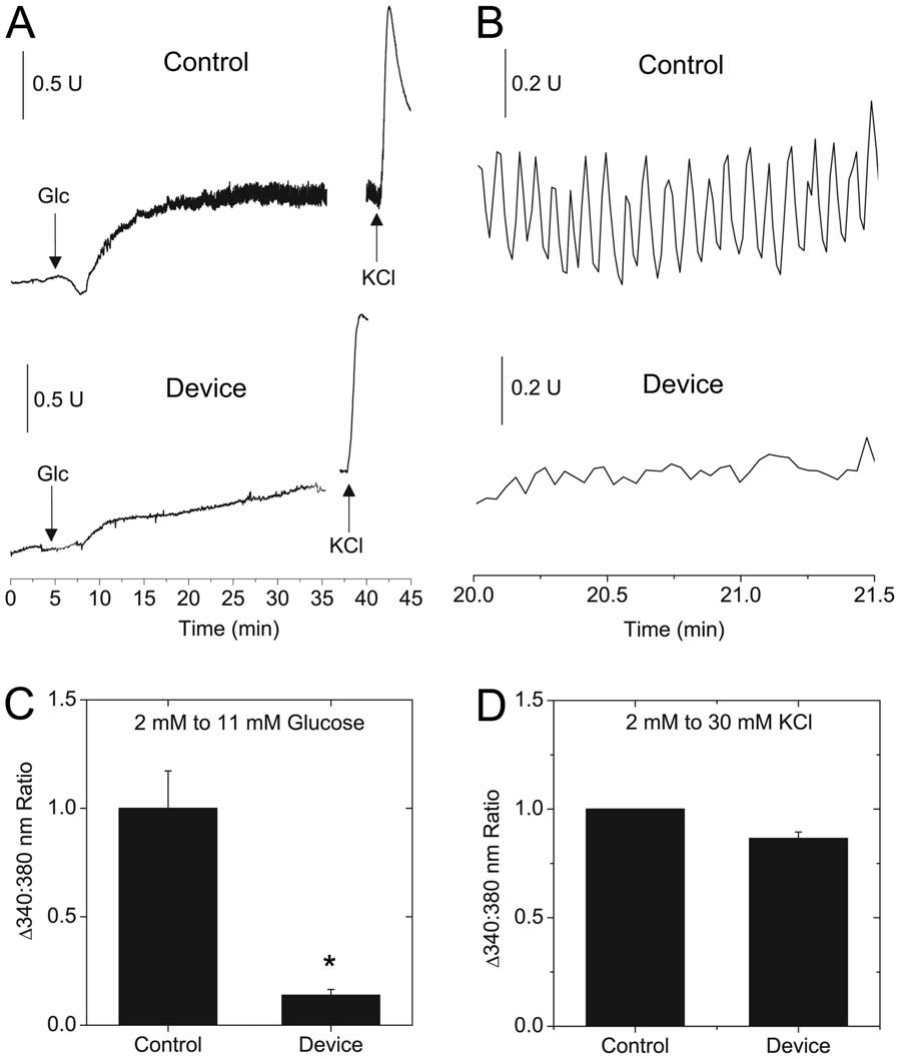
Measuring beta-cell [Ca^2+^]-activity using Fura-2. Islets subjected to 48 hr of fluid-flow (3 ml/hr) followed by labelling with Fura-2 to evaluate beta-cell [Ca^2+^]-responses. (A) Typical control and device-treated islets at 2 mM glucose displayed a consistent flat base-line [Ca^2+^]-response. After the addition of 11 mM glucose, control islets displayed normal [Ca^2+^]-oscillations while the device-treated response was small and flat. Data is representative of the response from a total of 11 islets from N = 3 mice, with the islets from each mouse isolated on independent days. (B) Control islets typically displayed fast Ca^2+^-oscillations at 11 mM glucose while the response of device treated islets was flat. (C) The change in 340∶380 nm ratio is the transition from 2 to 11 mM glucose in control and device-treated islets. (D) The change in 340∶380 nm ratio transitions from 11 mM glucose to 30 mM KCl in control and device-treated islets. The similar 30 mM KCl response delta indicates similar electrical activity in response to membrane depolarization. Each bar consists of the data collected from a total of at least 6 islets from 3 mice, with the islets from each mouse isolated on independent days. The * indicates p<0.05 compared to control by two-tailed student's t-test.

In our design, beta-cells on the periphery of the islet experience the greatest shear stress since they are subjected to the highest flow rates. To evaluate the functionality of the beta-cells of device-treated islets, we measured the intracellular [Ca^2+^]-response to glucose challenge and membrane depolarization using Fura-2 (**Fig. 5**). Fura-2 is an acetoxymethyl (AM) dye that due to restricted diffusion mainly reports on responses from peripheral cells of tissue. Both control and device-treated islets had low non-oscillatory [Ca^2+^]-responses at 2 mM glucose (340∶380 nm ratio), which is well below the normal response threshold (**Fig. 5A**). In contrast, control islets showed a significant rise in [Ca^2+^] at 11 mM glucose that was not present in device-treated islets (**Fig. 5A, B & C**), and both showed a consistent response to KCl-induced membrane depolarization (**Fig. 5A & D**).

**Figure 6:**
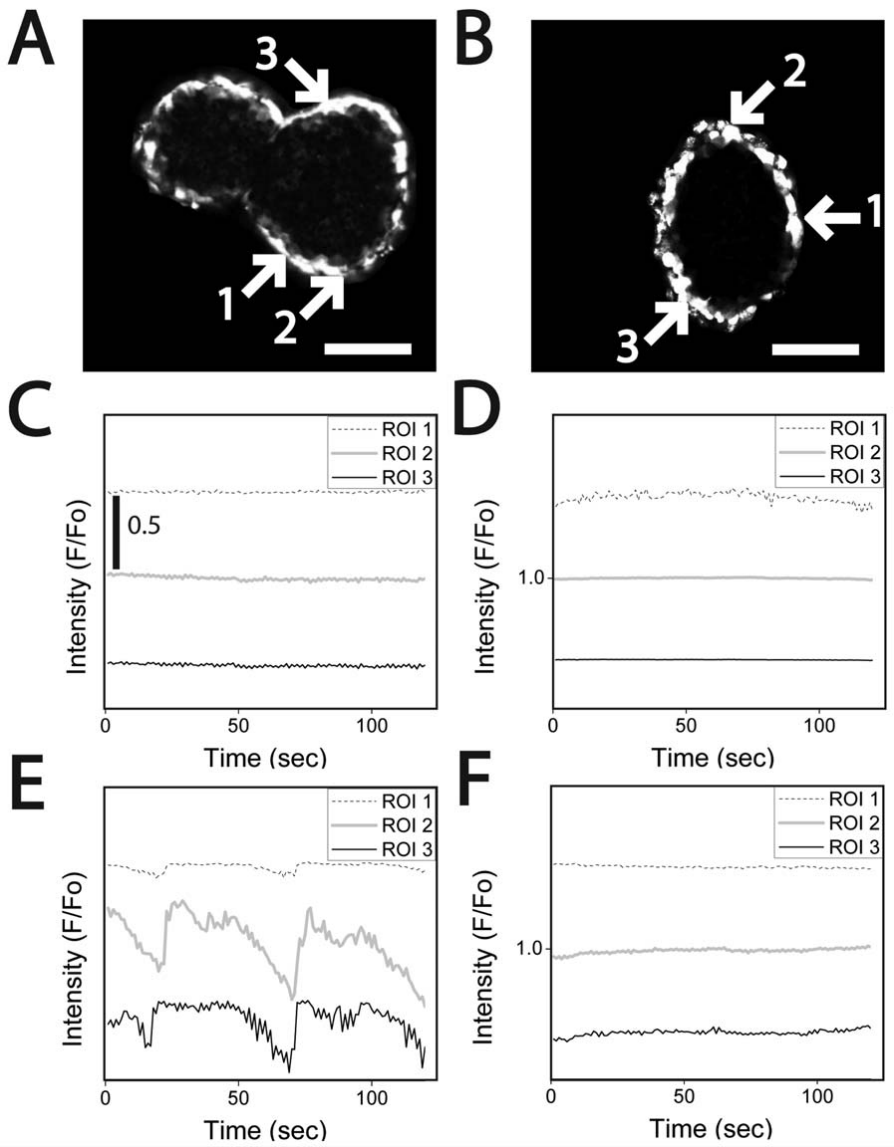
Monitoring synchronous beta-cell [Ca^2+^]-response in pancreatic islets using Fluo-4. Islets were cultured in control or microfluidic device flow (3 ml/24 hr) for 48 hr prior to labelling with Fluo-4. The dye labels cells as the periphery of the islet as observed using confocal microscopy by a ring of labelled cells. Representative control (*left*, A) and device-treated (*right*, B) islets are shown, each with three regions of interest (ROI) that are subsequently plotted in (C–F) (Scale bar 120 µm, 90 µm respectively). (C & D) At 2 mM glucose, the [Ca^2+^]-response is flat as expected in both the control (*left*, C) and device-treated islets (*right*, D). (E & F) At 11 mM glucose, [Ca^2+^]-oscillations were synchronous in functioning control islets (E, compare ROI 1, 2 and 3); however, no [Ca^2+^]-oscillations were observed in the device-treated islets (F) suggesting moderate damage to the periphery of the islet.

To measure [Ca^2+^]-oscillation synchrony, islets were labelled with Fluo-4 and imaged using a confocal microscope (**Fig. 6**) [17]. These cross-sectional images show that like other AM-dyes Fluo-4 only labels a few cell layers deep into tissue near the periphery (**Fig. 6A & B**). Three regions of interest containing multiple cells are shown in these representative images (**Fig. 6A & B** **,** ***arrows***) with normalized intensities (F/F_0_) plotted (**Fig. 6C–F**). At 2 mM glucose, control and device-treated islets showed a flat [Ca^2+^]-profile consistent with our Fura-2 data (**Fig. 6C & D**). In contrast and also consistent with our Fura2 data, 11 mM glucose caused oscillations only in the control islets (**Fig. 6E & F**). Overall, our data indicate a muted glucose-stimulated [Ca^2+^]-response from the periphery of device-treated islets, but that the islets are not damaged to the point of being electrically active at low glucose.

**Figure 7:**
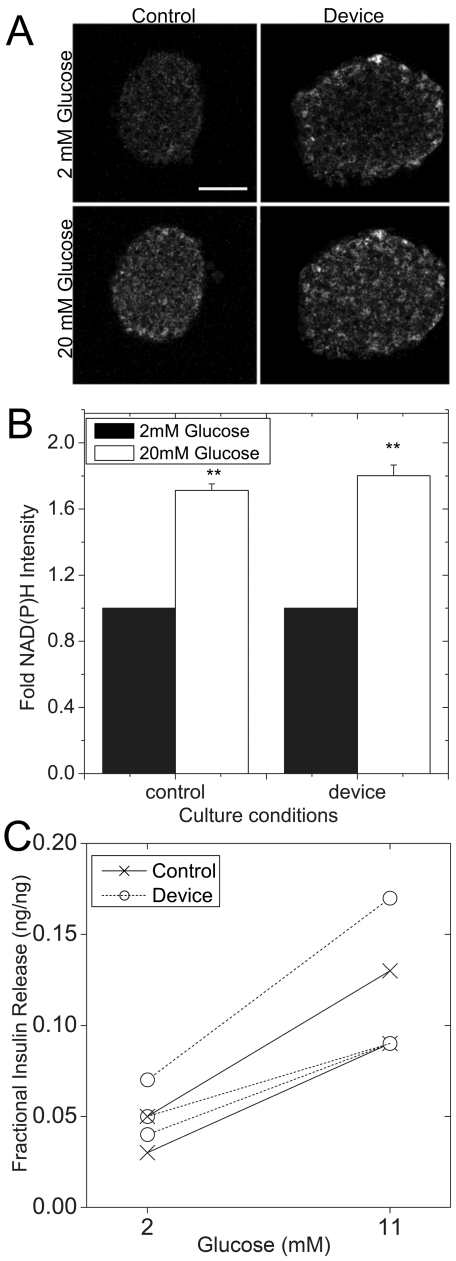
Glucose-stimulated NAD(P)H and insulin responses. Islets were cultured in control conditions or in microfluidic device flow (3 ml/24 hr) for 48 hr. (A) Control and flow treated islets were subsequently treated with 2 and 20 mM glucose and imaged using two-photon excitation of NAD(P)H. Scale bar in top left panel is 50 µm. (B) The fold NAD(P)H intensity from control and device treated islets. Data shown is the mean ± sem for 21 and 24 islets collected from 3 separate mice on independent days. The * indicates p<0.05 compared to control by two-tailed student's t-test. (C) Islets were cultured in control or in microfluidic device flow (device, 3 ml/24 hr) for 48 hr. The control islets were placed in a microfluidic device and both control and flow treated islets were subsequently treated with 2 mM glucose followed by 11 mM glucose in imaging buffer. The paired effluent was collected from each channel containing 4–6 islets. Insulin levels were then normalized to total insulin per channel using effluent collected with Triton X-100 permeabilization. A comparison between control and device-treated islets shows similar basal insulin levels with an increase in secretion at the higher glucose concentration. The data shown are from duplicate channels from 4 mice collected on independent days.

To investigate the activity of all the cells in the islet and not just those at the periphery, we imaged the glucose-stimulated metabolic response using two-photon imaging of the NAD(P)H response and also measured glucose-stimulated insulin secretion (**Fig. 7**). Two-photon imaging of NAD(P)H autofluorescence allows us to measure the redox state of living pancreatic islets with high spatial resolution [17], [20], [21], [22]. More specifically, islets cultured for 48 hr in control and microfluidic device-flow (3 ml/24 hr) were imaged in 2 and 20 mM glucose (**Fig. 7A**). The representative images were collected ∼20 µm deep into the tissue to provide a cross-section through the islet. Control islets in 2 mM glucose exhibit even autofluorescence across the tissue (**Fig 7A** **, top left**). In contrast, many of the device-treated islets at 2 mM glucose had higher intensity in the peripheral cells consistent with our previous experience that islets increase base line NAD(P)H when stressed (**Fig. 7A** **, top right**) [17]. Challenging the islets with glucose raised NAD(P)H intensity in both control and device-treated islets, consistent with a normal response in the majority of central beta-cells. On average, control and device-treated islets showed a similar ∼1.7-fold increase in NAD(P)H intensity (**Fig. 7B**). Importantly, these responses were collected from the central cells to avoid the brighter less responsive peripheral regions. To determine whether this metabolic response translates to normal overall insulin secretion, the glucose-stimulated insulin response was measured from control and device-treated islets (3 ml/24 hr) after 48 hr culture. This study used a single channel microfluidic device to allow analysis of the insulin in the effluent from islets subsequently stimulated with 2 mM and 11 mM glucose (**Fig. 7C**). The device-treated islets showed a steady rise in glucose-stimulated insulin secretion that was similar to control islets, suggesting a normal glucose-stimulated response. Overall, these data show that device-treated islets maintain their glucose-stimulated metabolic and insulin responses, and suggest that flow damage is limited to the peripheral cells.

## Discussion

Our data show that isolated pancreatic islets treated to media flow in a microfluidic device preserve EC density and connected length better than islets maintained in non-flowing traditional culture. Our data also show that the positive effect is due to intercellular flow improving serum albumin access to the center of the islet. While media flow dampened the glucose-stimulated [Ca^2+^]-response, this effect is limited to the peripheral beta-cells with normal glucose-stimulated two-photon NAD(P)H response and insulin secretion. Overall, these data indicate that microfluidic devices are a promising platform for long-term culture and maintenance of *ex vivo* pancreatic islets.

Previous studies used perfusion chambers to culture islets in laminar flow while sampling bulk O_2_ consumption rate [11], [12], [13]. In contrast, our setup allowed us to image the tissue using fluorescence microscopy (PECAM-1 immunofluorescence, Ca^2+^-response, two-photon NAD(P)H) and examine the effect of media flow on the ECs of individual islets. The ECs of mature blood vessels have a characteristic cobblestone morphology associated with tight cell-cell junctions [9], [27]. This morphology and cell-cell junction formation is a critical regulator of EC growth and apoptosis [7], [28]. We and others have shown that EC morphology in culture slowly deteriorates with cells initially coalescing followed by a reduction in overall number over a period of days. In contrast, device-treated islets maintained significantly greater EC area and connected length compared to control islets.

We narrowed the effect of microfluidic device culture to improved access of serum albumin through the intercellular spaces. Individual rat islets *in vivo* are normally exposed to ∼0.03 ml/24 hr of blood containing serum albumin [24], [25]. Serum albumin is an anti-apoptotic signal for ECs that has restricted diffusion in tissue due to a large hydrodynamic radius. In the absence of flow, access of serum albumin depends solely on diffusion. Our data are consistent with flow in the device overcoming limited diffusion to stimulate the ECs within the tissue. We also observed a significant reduction in necrosis in flow-treated islets. It should be noted that even with serum albumin gaining access, we only partially maintained EC morphology with significant loss in density and length still observed compared to freshly isolated islets. To improve these responses, we propose further optimization of our media including addition of angiogenic factors that may have previously shown limited effect due to poor tissue penetration.

Although we used linear flow velocities (2–6 cm/min) similar to other perfusion columns, our microfluidic device has a significantly smaller cross-sectional area (0.04 mm^2^ vs. 4.9–55 mm^2^) [11], [12], [13]. This creates a much higher fluidic resistance down the channel. By calculating the fluidic-resistance the length of a typical islet (100 µm), we estimate that islets experience a drop in pressure of ∼10^−5^ kPa along their length when cultured at 3 ml/24 hr. This drop forces media to flow through the islet rather than simply around it consistent with improved serum albumin access and reduced necrosis. In flow greater than 3 ml/24 hr, media exchange inside the islet occurred in less than 30 s. However, we observed an asymptotic increase in the time to clear the islet below 3 ml/24 hr indicating we are at the threshold pressure required to induce significant intercellular flow. In contrast, we estimate that in larger perfusion columns with 4.9–12.6 mm^2^ cross sectional area, islets experience 200 to 5000-fold smaller pressure drop across the islet and are therefore not likely to induce significant flow in the intercellular space. More recently, microfluidic devices have been designed for short-term culture prior to transplantation; however, these devices are specifically designed to hold the islets in a reservoir with minimal flow and likely do not cause significant intercellular media-flow [29].

Beta-cells respond to glucose through metabolic closure of ATP-sensitive potassium channels (K_ATP_), membrane depolarization, [Ca^2+^]-influx through voltage gated calcium channels, and insulin secretion [30]. We examined the glucose-stimulated [Ca^2+^]-response using two dyes, Fura-2 and Fluo-4, and observed a robust [Ca^2+^]-response to membrane depolarization. However, the glucose-stimulated response of device-treated islets was muted in comparison to control islets. We further examined the glucose-stimulated metabolic response using two-photon excitation of NAD(P)H autofluorescence. This technique provided a quantitative measure of the glucose-stimulated metabolic response with sufficient spatial resolution to determine the effect of flow on both peripheral and central beta-cells. Consistent with the muted [Ca^2+^]-response, the peripheral cells showed a muted glucose-stimulated NAD(P)H response. However, consistent with a normal response from the remainder of beta-cells, the central cells of device-treated islets had similar glucose-stimulated NAD(P)H responses compared to control islets. Finally, device-treated islets had similar glucose-stimulated insulin secretion compared to control islets. Overall, our data suggest the majority of central beta-cells maintain glucose-stimulated responses in the microfluidic device, with minor damage to the peripheral cells.

We are particularly interested in using microfluidic devices to study the communication between beta-cells and ECs via extracellular matrix (ECM) deposition and remodeling. ECs are primarily responsible for deposition of islet ECM, which directly affects beta-cells by modulating cell survival, proliferation, differentiation and insulin secretion through a number of mechanisms [31], [32]. We recently showed that beta-cell fibroblast growth factor receptor-1 (FGFR1) expression and signaling are altered by the ECM, suggesting activity can be modulated by EC-interaction to effect proliferation and glucose-stimulated insulin secretion [33]. Our microfluidic platform enhances the culture lifespan of islet associated ECs, providing a controlled environment to study beta-cell-EC interaction.

Our results are also relevant to the treatment of type 1 diabetes through transplantation of pancreatic islets following the Edmonton Protocol [34]. This treatment has limited initial and long-term efficacy with only 58% of patients gaining insulin independence and 76% of patients requiring insulin after two years [35]. We propose to improve revascularization efficiency by maintaining the ECs during culture or alternatively ‘priming’ them prior to transplantation. The flexibility of microfluidic device design allows scale-up in the number of islets needed for transplantation (i.e. increasing channel number). However, scaled-up versions will need to maintain high pressure differential along the channel in combination with low linear velocity. These studies will also need to examine the effect of flow on the peri-islet capsule as it has been postulated to act as a barrier to the immune response [36].

## Supporting Information

Figure S1 **Custom microfluidic devices to culture** ***ex vivo*** **pancreatic islets in laminar flow.** (A) A three channel microfluidic device composed of PDMS, glass coverslip, and inlet- and outlet-tubing. This device is designed to supply flow at varied rates in the three independent channels. Bromophenol blue dye is used to highlight the three separate channels with a common output. (B) The flow-rate in each channel is determined by its resistance, which is directly related to channel length. Each channel has a unique input tubing, main channel, and dam structure that is followed by an output channel of different length. The outlet channels are ultimately connected to a single outlet tube. This places a similar pressure differential (air to syringe pump) across the different channels of the device. (C) By using a single syringe pump, fluid is pulled through each channel at a different rate during culture. This results in fluid being pulled from the syringe reservoirs at different rates. The reservoir height (before and after culture) was ultimately used to determine the average flow-rate during culture in each channel. (D) The normalized values of the actual flow rate (black bars) and calculated relative resistance (open bars) of each channel are shown in the absence of islets (N = 5). (E & D) The design is simplified to contain single independent channels with dam structures. The input and output channels are identical in length. This design provides multiple channels for high throughput/parallel culture and limits loss due to air bubble occlusion. (Scale bar A = 10 mm, B = 6 mm, C = 15 mm, E = 10 mm, F = 2 mm).
(TIF)

Figure S2 **A schematic of our process and the actual bench-top microfluidic incubator.** (A) A schematic representation shows the variation of temperatures across the setup. The syringe reservoir and microfluidic device are maintained at 44°C and 37°C, respectively. These temperatures take advantage of the lower dissolvability of air in water at higher temperatures to slightly degas the media prior to entering the microfluidic channels. (B) A corresponding picture of the microfluidic device setup shows two hotplates to control water bath temperature, one with a thermocouple to guarantee a constant 37°C. Media perfusion is accomplished with a single syringe pump (three channel device) or multiple pumps (independent channel device). The device and media reservoirs are submerged in their respective water baths. The media in the syringe is capped with ∼1 cm of mineral oil to limit pH drift and evaporation, and the top of the syringe reservoirs are covered with AirPore tape (3 M). Both beakers are covered with plastic wrap to reduce evaporation from the open water baths.
(TIF)

Figure S3 **Measuring beta-cell physiology using an independent channel microfluidic device.** (A) The device shown has four identical flow channels, and a secondary layer of PDMS used to form on-chip wells for easy reagent switching. Islets are cultured in this device as described previously followed by subsequent removal of the input tubing. The removal of this tubing starts flow from the on-chip wells with flow controlled by the syringe pump connected to the output tubing (B) Islets were labelled with Fura-2 in the device (4 µM, 200 µL/mL, 1 hr) and transferred to the microscope stage for imaging. The transition from 2 to 11 mM glucose is accomplished by changing the ∼200 µL on-chip well. Islets in 11 mM glucose displayed a normal [Ca^2+^]-response. The addition of 30 mM KCl showed a larger [Ca^2+^]-spike consistent with membrane depolarization. Note that the intervals of specific reagents are indicated with a straight line (2–4 min, 36–38 min).
(TIF)

Text S1 **Supplemental text.** Detailed description of the microfluidic device design and process.
(DOC)
