# Supplementary figures and images for: Culturing Pancreatic Islets in Microfluidic Flow Enhances Morphology of the Associated Endothelial Cells
Source: PLoS One. 2011 Sep 22;6(9):e24904. doi: 10.1371/journal.pone.0024904 (PMC3178551; doi:10.1371/journal.pone.0024904)

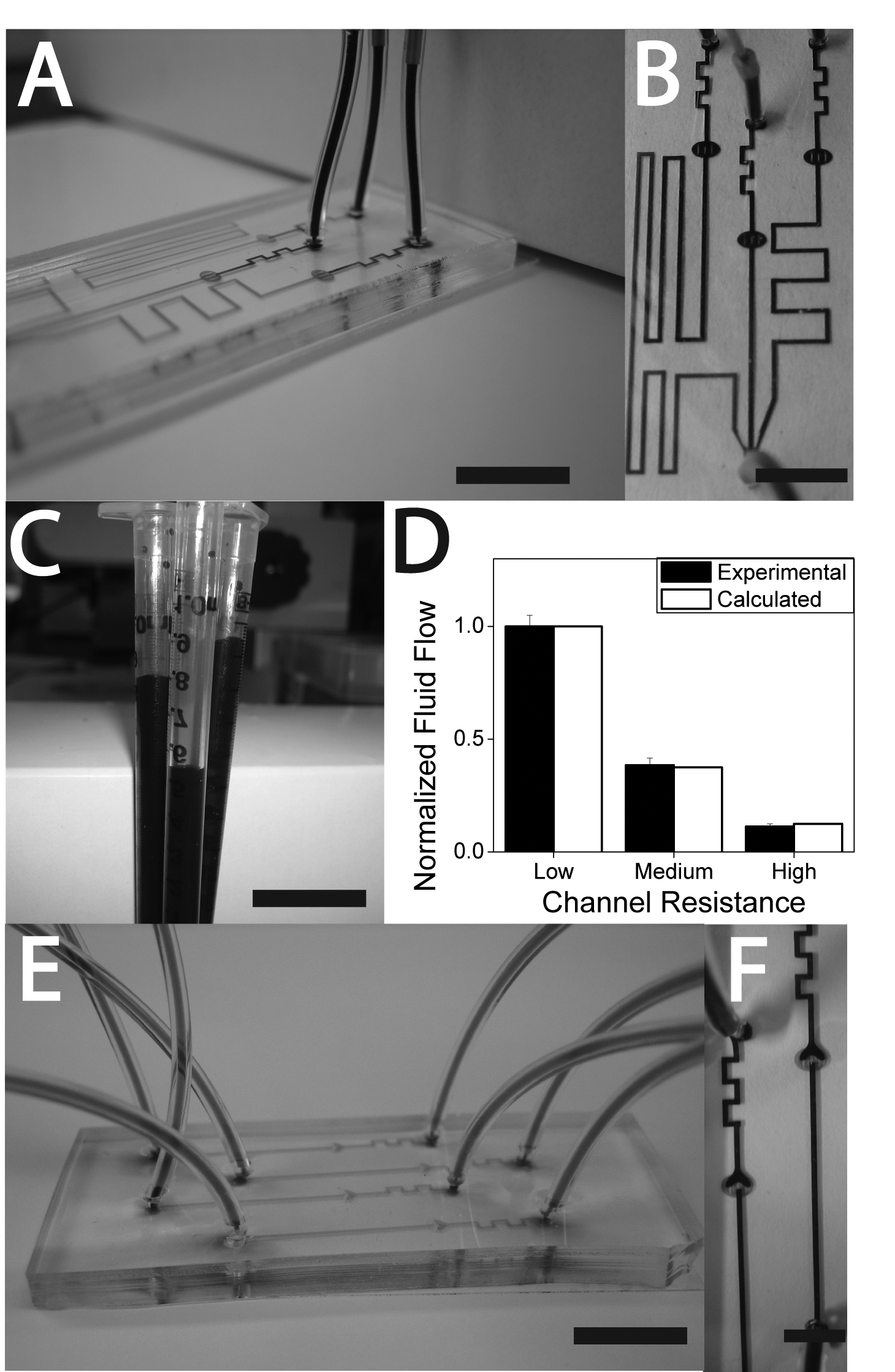

Supplement: Figure S1 — Custom microfluidic devices to culture ex vivo pancreatic islets in laminar flow. (A) A three channel microfluidic device composed of PDMS, glass coverslip, and inlet- and outlet-tubing. This device is designed to supply flow at varied rates in the three independent channels. Bromophenol blue dye is used to highlight the three separate channels with a common output. (B) The flow-rate in each channel is determined by its resistance, which is directly related to channel length. Each channel has a unique input tubing, main channel, and dam structure that is followed by an output channel of different length. The outlet channels are ultimately connected to a single outlet tube. This places a similar pressure differential (air to syringe pump) across the different channels of the device. (C) By using a single syringe pump, fluid is pulled through each channel at a different rate during culture. This results in fluid being pulled from the syringe reservoirs at different rates. The reservoir height (before and after culture) was ultimately used to determine the average flow-rate during culture in each channel. (D) The normalized values of the actual flow rate (black bars) and calculated relative resistance (open bars) of each channel are shown in the absence of islets (N = 5). (E & D) The design is simplified to contain single independent channels with dam structures. The input and output channels are identical in length. This design provides multiple channels for high throughput/parallel culture and limits loss due to air bubble occlusion. (Scale bar A = 10 mm, B = 6 mm, C = 15 mm, E = 10 mm, F = 2 mm). (TIF) [file pone.0024904.s001.tif]

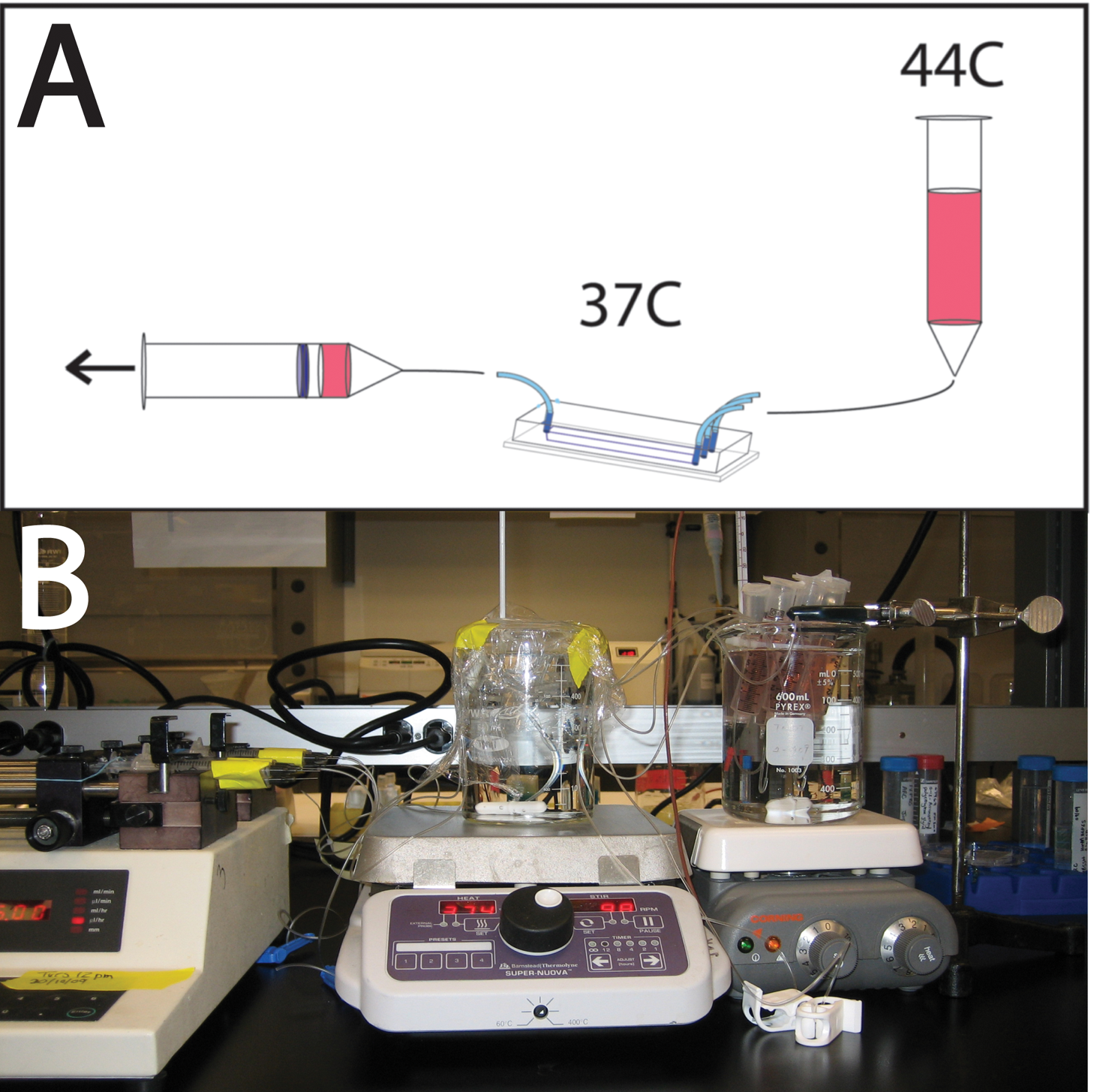

Supplement: Figure S2 — A schematic of our process and the actual bench-top microfluidic incubator. (A) A schematic representation shows the variation of temperatures across the setup. The syringe reservoir and microfluidic device are maintained at 44°C and 37°C, respectively. These temperatures take advantage of the lower dissolvability of air in water at higher temperatures to slightly degas the media prior to entering the microfluidic channels. (B) A corresponding picture of the microfluidic device setup shows two hotplates to control water bath temperature, one with a thermocouple to guarantee a constant 37°C. Media perfusion is accomplished with a single syringe pump (three channel device) or multiple pumps (independent channel device). The device and media reservoirs are submerged in their respective water baths. The media in the syringe is capped with ∼1 cm of mineral oil to limit pH drift and evaporation, and the top of the syringe reservoirs are covered with AirPore tape (3 M). Both beakers are covered with plastic wrap to reduce evaporation from the open water baths. (TIF) [file pone.0024904.s002.tif]

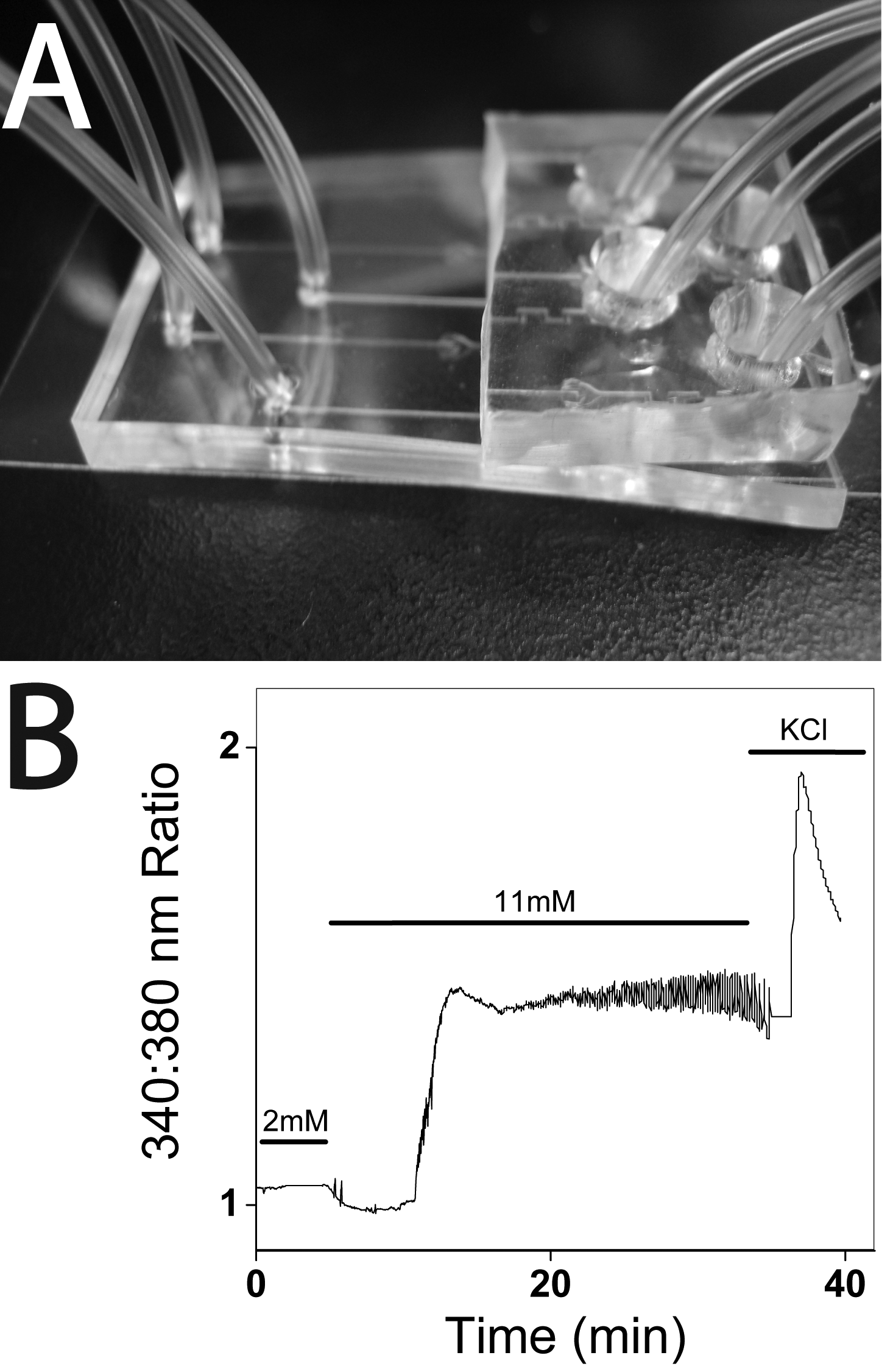

Supplement: Figure S3 — Measuring beta-cell physiology using an independent channel microfluidic device. (A) The device shown has four identical flow channels, and a secondary layer of PDMS used to form on-chip wells for easy reagent switching. Islets are cultured in this device as described previously followed by subsequent removal of the input tubing. The removal of this tubing starts flow from the on-chip wells with flow controlled by the syringe pump connected to the output tubing (B) Islets were labelled with Fura-2 in the device (4 µM, 200 µL/mL, 1 hr) and transferred to the microscope stage for imaging. The transition from 2 to 11 mM glucose is accomplished by changing the ∼200 µL on-chip well. Islets in 11 mM glucose displayed a normal [Ca2+]-response. The addition of 30 mM KCl showed a larger [Ca2+]-spike consistent with membrane depolarization. Note that the intervals of specific reagents are indicated with a straight line (2–4 min, 36–38 min). (TIF) [file pone.0024904.s003.tif]
