# Supplementary material for: Culturing Pancreatic Islets in Microfluidic Flow Enhances Morphology of the Associated Endothelial Cells
Source: PLoS One. 2011 Sep 22;6(9):e24904. doi: 10.1371/journal.pone.0024904 (PMC3178551; doi:10.1371/journal.pone.0024904)
Supplement: Text S1 — Supplemental text. Detailed description of the microfluidic device design and process. (DOC) [file pone.0024904.s004.doc]

*Microfluidic Device Design:* Our goal was to study the effect of laminar media flow on *ex vivo* pancreatic islets. We anticipated that flow in these devices would increase media exchange to the center of the islet and possibly induce pro-survival shear stress to the associated endothelial cells [1].

To examine the effect of flow on the ECs of *ex vivo* islets, we designed both a three-channel and an independent-channel microfluidic device (**Fig. S1**). Islets were brought into the 125 μm tall microfluidic channels through inlet tubing [2,3]. The islets traveled freely down this channel until blocked by a 25 μm tall dam structure. The dam structure allowed solution to flow past, but blocked the movement of islets, which are generally 100 μm or more in diameter. The islets were therefore held stationary in laminar flow that provided enhanced media exchange and shear stress.

We fabricated our devices using molded polydimethylsiloxane (PDMS) bonded to a No. 1 glass cover-slip (**Fig. S1A**). PDMS is the predominant polymer used for soft lithography due a number of properties including transparency, low cost, and quick manufacturing process [4]. PDMS is also inherently permeable to gas, and therefore compatible with living samples [5]. Due to this permeability, researchers often go to extreme lengths to induce hypoxia in these devices, for example, by coating the PDMS in paralene C [6]. However, PDMS permeability adds complexity by supporting formation and expansion of air bubbles inside the microfluidic device. A No. 1 glass cover-slip provides optimal thickness (0.17 mm) when working the high numerical aperture (NA) lenses, and therefore allows superior fluorescence signal collection and resolution.

The three channel microfluidic device was designed to provide a wide range of flow rates on a single chip using a single syringe pump (**Fig. S1A and B**). In designing this device, we aimed for a 10-fold difference in flow-rates across the channels to reflect the wide response range of cells to shear [7]. Each channel had an independent inlet tube followed by main channel and dam structure identical to the other channels in the device. The dam structures were subsequently followed by channels of different length, each ultimately connected to a common outlet-tube. This design placed an identical pressure differential (open-air to syringe pump) across each of the variable channels. The flow rate varies in each channel due to resistance:

$Q=\frac{\Delta P}{R}$ (1)

where, Q is the flow rate, ∆P is the change in pressure along the channel (open-air to syringe pump), and R is the fluidic resistance. By using open inlet reservoirs and a single syringe pump, our design placed similar pressure across each channel making the flow-rates in each channel proportional to the resistance.

The resistance of each channel can be calculated using the common resistance formula (1) [8,9],

$R=\frac{12\mu L}{wh^{3}}\left[1-\frac{h}{w}\left(\frac{192}{\pi^{5}}\sum_{n=1,3,5} \frac{1}{n^{5}}\tanh\left(\frac{n\pi w}{2h}\right)\right)\right]^{-1}$ (2)

where, R was the total resistance of a channel with a single width and height. L was the channel length. With w>>h, the total resistance calculation is further simplified to the following:

$R=\frac{12\mu L}{wh^{3}}$ (3)

For a circular channel the total resistance calculation is:

$R=\frac{8\mu L}{\pi\cdot r^{4}}$ (4)

Each channel of our device contained three sections: a main channel, a dam structure, and a variable-length channel. Since these sections are in series, the total resistance in a channel is determined by adding the resistance of the individual sections. The main channel was 125 μm tall and 300 μm wide, and 1.2 cm in length from the inlet tubing to the dam structure. The dam structure was 25 μm tall and 1,800 μm wide, but only 0.13 cm long. The outlet channels were 125 μm tall and 300 μm wide, and 1.5, 4, or 12 cm in length. With identical channel dimensions for the first two sections, the relative resistance was essentially determined by the length of the variable-length outlet channel. When normalized to the channel with the shortest length and lowest resistance (**Fig. S1A and B, center channel**), we expected to achieve a flow-rate ratio of 1: 3/8: 1/8. In other words, by using a single syringe pump we expected to pull media from the three reservoirs and past the islets at three different rates. As fluid flow rate is directly proportional to shear stress, this device also had the potential to treat islets to varied shear stress.

To test the actual flow rates in our device, we measured the input reservoir heights before and after flow (**Fig. S1C**). Consistent with the intended design, the observed and calculated flow rates showed increasingly less flow in the low, medium and high resistance channels, respectively (**Fig. S1D**). It is important to note that throughout all of our experiments we determined the average flow rate during culture by measuring changes in the reservoir syringe. Overall, these data show that the device reliably drew fluid spanning a nearly 10-fold flow-rate, and were therefore well suited to optimize long-term culture.

Upon optimization of the flow rates, we subsequently used a microfluidic device with independent channels that was simpler and better suited for subsequent live cell imaging or assay (**Fig. S1E-F**). Like the previous design, the independent channels had a main channel followed by a dam structure, but now the outlet was identical in length across the device with independent reservoir and syringe tube. This design allowed us to treat islets in parallel while limiting failure to a single channel during culture (air bubble occlusion or otherwise). This design also facilitated subsequent assay of the tissue using live cell imaging or other assays.

*Bench-top incubator:* We aimed to culture *ex vivo* islets inside our device over a period of days with media maintained at proper physiological temperature, salinity, and pH. One solution was to place the entire setup inside a CO_2_ incubator; however, few labs have sufficient room inside their incubator to house the device, tubing, and syringe pumps. We instead created an incubator-like environment on the bench-top (**Fig. S2**). This setup mimicked the incubator environment; however it also addressed two inherent issues when using microfluidic devices: occlusion of the channels by air bubbles, and maintenance of pH and salinity in an open-reservoir design.

Occlusion of microfluidic channels with air bubbles is a terminal problem in biological applications as it can alter fluid flow patterns, occlude ports, and damage a biological sample [10]. A common solution to reduce the effect of bubbles is to use a bubble trap [11]; however, this structure adds further complexity to immunofluorescence labelling and live cell imaging/assays. One source of air bubbles is from media degassing as it enters the microfluidic channels from the reservoir. To reduce this effect, we leveraged the varied dissolvability of gas in media at different temperatures (**Fig. S2A**). Media is often heated from room temperature (RT) to 37 °C as it enters the microfluidic device. The dissolvability of air in water decreases from ~22 μg/mL at RT to ~19 μg/mL at 37 °C, therefore, 1 mL of solution will release ~20 μL of gas as it is pulled from a RT-reservoir into the 37 °C device [11]. Our solution was to heat the media reservoir slightly (44 °C) to reduce the dissolvability of air to 14 μg/ml [12]. The media will reabsorb air as it travels through the channels due to the permeability of air through PDMS and also reabsorb air from bubbles to reduce their size and number.

Air bubbles can also be drawn into the device from the input media or around the access tubing. To reduce the number of bubbles drawn into the device from the air, we tapped the syringe prior to attaching tubing to dislodge any bubbles near the tip and submerged the device in water (**Fig. S2B**). After loading freshly isolated islets into the device and connecting tubing to the reservoir syringes, the devices were also submerged ~2 cm deep in a stirred 37 °C hot water bath (**Fig. S2B**) [13,14]. The water bath kept the entire device at a constant 37 °C. Overall, by increasing the temperature of the reservoir-media and submerging the device we successfully ran our devices for 24-48 hr without bubble occlusion in nearly 90% of channels.

In this setup, the reservoir syringes were covered with air pore paper, which leaves the reservoir open to air, but prevents evaporation and bacterial growth contamination (3M). As described, the reservoir syringe was heated in a water-filled beaker on a hotplate and monitored with a thermometer. We controlled the temperature of the water bath using a thermocouple connected to the hot plate; however our lab has also successfully used submersion in a standard heated water bath. Finally, the output tube from each device was attached to a syringe pump, the hot water bath was covered with cellophane to prevent evaporation, and the output syringe pump established the desired flow rate to run for 24-72 hr.

In a standard cell incubator, the pH of the media is normally buffered by the 5% CO_2_ atmosphere of the incubator. In a bench-top environment with open-well configuration, the pH of the media slowly increases and will eventually become hostile for the tissue. This change in pH is a direct result of the diffusion of CO_2_ from the media to the surrounding air. This effect is further exacerbated with decreased solubility of CO_2_ in the media reservoir at 44°C (~56% decrease of CO_2_ / H_2_O vol/vol) [15]. An incubator eliminates this problem by maintaining an ambient 5% CO_2_ atmosphere. To limit changes in pH, we effectively slowed the diffusion of CO_2_ from the media by adding ~1 cm of mineral oil to the top of the media in the reservoir (data not shown) [16,17,18]. The mineral oil cap also further prevents evaporation of the media, reducing subsequent increases in salinity.

Our bench-top setup provides the necessary requirements of an incubator with controlled temperature and pH, and with the added benefit of reduced size. The entire setup required only a few square feet of counter space with available wall sockets. Each component could be moved conveniently as required. This modularity allowed for changes in equipment setup and was particularly suited for subsequent live cell imaging. Having the opportunity to remove the device to place it in a heated microscope stage for live cell imaging with relative ease was essential for measuring the molecular physiology of the tissue post-culture. This modularity also facilitated subsequent immunofluorescence imaging and live cell imaging/analysis. Overall, we found using a bench-top incubator retained the benefits of a traditional cell culture incubator with many added benefits including portability, increased flexibility, compatibility with imaging techniques, and reduced reagent use.

*Live cell analysis:* In addition to fixed labelling and imaging after extended culture, we found our device and setup was flexible to live cell imaging (**Fig. S3**). To transition from long-term culture to analysis of live cell responses, we included a second layer of PDMS with on-chip reservoirs (**Fig. S3A**). The two pieces of PDMS were irreversibly bonded using plasma oxidation [19]. Islets were initially cultured in this device (as described previously) using a larger syringe reservoir submerged in a 44 °C water bath. The tubing was subsequently removed from the device leaving the ~200 μL on-chip reservoir, which was ideal for quick exchange of solutions during live cell imaging.

The beta cells of pancreatic islets respond to blood glucose through the regulated secretion of insulin. A glucose concentration greater than 7 mM leads to an increase in intracellular calcium ([Ca^2+^]) and insulin secretion [20]. To measure the glucose-stimulated [Ca^2+^]-response, we imaged islets labelled with Fura-2 (4 μM, 1-2 hr) sequentially treated with low and high glucose as well as membrane depolarization (**Fig. S3B**). Control islets in the device showed a non-oscillatory [Ca^2+^]-response at 2 mM glucose (340:380 nm ratio), which is well below the normal response threshold. Islets also showed a significant [Ca^2+^]-response with oscillations at 11 mM glucose and an even larger influx with KCl-induced membrane depolarization. Overall, we were able to culture islets in our device and transition to live cell imaging using this modified design.

**References**

1. Dimmeler S, Haendeler J, Rippmann V, Nehls M, Zeiher AM (1996) Shear stress inhibits apoptosis of human endothelial cells. Febs Letters 399: 71-74.

2. Rocheleau JV, Remedi MS, Granada B, Head WS, Koster JC, et al. (2006) Critical role of gap junction coupled KATP channel activity for regulated insulin secretion. Plos Biology 4: e26.

3. Rocheleau JV, Walker GM, Head WS, McGuinness OP, Piston DW (2004) Microfluidic glucose stimulation reveals limited coordination of intracellular Ca2+ activity oscillations in pancreatic islets. Proceedings of the National Academy of Sciences of the United States of America 101: 12899-12903.

4. McDonald JC, Whitesides GM (2002) Poly(dimethylsiloxane) as a material for fabricating microfluidic devices. Accounts of Chemical Research 35: 491-499.

5. Morisato A, Pinnau I (1996) Synthesis and gas permeation properties of poly(4-methyl-2-pentyne). Journal of Membrane Science 121: 243-250.

6. Oppegard SC, Blake AJ, Williams JC, Eddington DT Precise control over the oxygen conditions within the Boyden chamber using a microfabricated insert. Lab Chip 10: 2366-2373.

7. Duan Y, Gotoh N, Yan Q, Du Z, Weinstein AM, et al. (2008) Shear-induced reorganization of renal proximal tubule cell actin cytoskeleton and apical junctional complexes. Proceedings of the National Academy of Sciences of the United States of America 105: 11418-11423.

8. Beebe DJ, Mensing GA, Walker GM (2002) Physics and applications of microfluidics in biology. Annual Review of Biomedical Engineering 4: 261-286.

9. Sia SK, Whitesides GM (2003) Microfluidic devices fabricated in poly(dimethylsiloxane) for biological studies. Electrophoresis 24: 3563-3576.

10. Meldrum DR, Holl MR (2002) Microscale bioanalytical systems. Science 297: 1197-1198.

11. Sung JH, Shuler ML (2009) Prevention of air bubble formation in a microfluidic perfusion cell culture system using a microscale bubble trap. Biomedical Microdevices 11: 731-738.

12. ToolBox TE (Retrieved 2010-03-15) Air Solubility In Water. The Engineering ToolBox.

13. Kadohama T, Nishimura K, Hoshino Y, Sasajima T, Sumpio BE (2007) Effects of different types of fluid shear stress on endothelial cell proliferation and survival. Journal of Cellular Physiology 212: 244-251.

14. Whitesides GM (2006) The origins and the future of microfluidics. Nature 442: 368-373.

15. Lange NA (1967) Lange's Handbook of Chemistry: McGraw-Hill.

16. Brown LF, Trlica MJ (1977) Interacting effects of soil-water, temperature and irradiance on co2 exchange-rates of 2 dominant grasses of shortgrass prairie. Journal of Applied Ecology 14: 197-204.

17. de Sousa CAF, Sodek L (2003) Alanine metabolism and alanine aminotransferase activity in soybean (Glycine max) during hypoxia of the root system and subsequent return to normoxia. Environmental and Experimental Botany 50: 1-8.

18. Espie G, Colman B (1987) The Effect of pH, O2, and Temperature on the CO2 Compensation Point of isolated Asparagus Mesophyll Cells. Plant Physiology 83: 113-117.

19. Duffy DC, McDonald JC, Schueller OJA, Whitesides GM (1998) Rapid prototyping of microfluidic systems in poly(dimethylsiloxane). Analytical Chemistry 70: 4974-4984.

20. Dean PM, Matthews EK (1970) GLUCOSE-INDUCED ELECTRICAL ACTIVITY IN PANCREATIC ISLET CELLS. Journal of Physiology-London 210: 255-&.
